# How and why pharmaceutical reforms contribute to universal health coverage through improving equitable access to medicines: a case of Ghana

**DOI:** 10.3389/fpubh.2023.1163342

**Published:** 2023-07-07

**Authors:** Augustina Koduah

**Affiliations:** Department of Pharmacy Practice and Clinical Pharmacy, School of Pharmacy, College of Health Sciences, University of Ghana, Accra, Ghana

**Keywords:** access to medicines, universal health coverage, policy prioritization, reforms, Ghana

## Abstract

**Background:**

Examining how and why a country prioritizes and implements pharmaceutical reforms tends to show complex processes and myriad efforts made toward improving access to medicines. This study examines factors that enabled the prioritization and implementation of selected pharmaceutical reform items and how these factors contributed to improving equitable access to medicines and universal health coverage in Ghana.

**Methods:**

An analytical framework was developed to identify variables to explore in answering the study questions and frame the analysis and presentation of findings. Documents analyzed included the National Medicines Policies, Health Sector Program of Work, and other health policies. Quantitative data were sourced from databases maintained by World Health Organization and the Institute for Health Metrics and Evaluation.

**Results:**

The three main factors, evidence, financial and technical support, and alignment to national and global policies, influenced the prioritization and implementation of access to medicines reforms. The reforms targeted rational selection and use of medicines, medicine pricing, sustainable medicine financing, and regulatory and supply chain systems. Although there were limited quantitative data to quantify access to medicine policies” impact on universal health coverage, it can be reasonably assumed that, in Ghana, access to medicine policies has contributed to financial protection and improved access to quality health services.

**Conclusion:**

Access to medicine policies targeted at promoting rational medicine selection and use, regulating medicine pricing and improving sustainable financing for medicines as well as the regulatory and supply chain systems arguably contributed to the attainment of UHC and must be sustained. Therefore, data collection and reporting indicators for access to medicines must be prioritized.

## Introduction

Access to safe, effective, quality, and affordable essential medicines by the population contributes to universal health coverage (UHC). Essential medicines are those that satisfy the priority health needs of the population (1), and as noted in Sustainable Development Goals (SDGs) 3.8, governments must seek to achieve universal health coverage for their population, including improving access to essential medicines and vaccines for all (2). Access to essential medicines is a central component of universal health coverage (3). Universal health coverage “*means that all people have access to the full range of quality health services they need, when and where they need them, without financial hardship”*. UHC seeks to improve access to quality health services, including essential medicines, by providing financial risk protections and increasing population coverage (4).

The World Health Organization (WHO) proposes four dimensions for improving access to medicines: (1) rational selection and use, (2) affordable prices (3), sustainable financing, and (4) reliable health and supply system (1). The rational selection and use of essential medicines include the development of evidence-based standard treatment guidelines and corresponding essential medicines list (EML) and its use for prescribing, dispensing, procurement, reimbursement, and training across the health sector. The rational selection and use of medicines contribute to improved access to quality health services and population coverage. The affordable price dimension deals with medicine pricing policies, such as promoting the use of generics, local manufacturing of medicines, markup regulations across the supply chain, pooled procurement, and tax exemptions for pharmaceutical products, and these policies contribute to reducing financial risk as medicines are made more affordable. The sustainable financing dimension deals with the reduction of out-of-pocket expenditures, increasing funding for essential medicines, and expanding health insurance, thus providing financial risk protection for the general population, especially the poor, and vulnerable population groups. The reliable health and supply system dimension deals with the regulation of medicines, integrating medicines in health sector developments and plans, and the supply of medicines and aims to ensure the population's essential medicines needs are covered (1, 5).

Improving access to medicines for all is complex and can be challenging. Globally and nationally, there are gains in the development of national treatment guidelines and essential medicine list (6–8); however, there are gaps at the national level in the selection of medicines compared with those recommended by the World Health Organization (7), and there are also reported cases of inappropriate use of medicines (9–12). The prices of medicines are high, making them inaccessible to, especially, the poor and vulnerable population groups (10–13). Effective public procurement systems are critical for ensuring access to medicines, but there are also reported challenges including the operations of many “middlemen” along the pharmaceutical supply chain (14) and shortage of medicines (10–12, 15).

In Ghana, access to essential medicines by the population is uneven (16) and hence uneven universal health coverage. Over the years, the government through the Ministry of Health (MoH) and its agencies prioritized and implemented pharmaceutical reform items to promote rational medicine selection and use, regulate medicine pricing, and improve medicine financing and the regulatory and supply chain systems (10–12). These pharmaceutical reform items were first noted in 1999 in the National Drug Policy, which aims “*to make essential drugs available and accessible to the population and ensure the safety, efficacy and the quality of drugs and their rational use by prescribers, dispensers, and consumers”* (12). It is important to understand factors influencing government prioritization and implementation of pharmaceutical reform items and how these reforms contributed to improving access to medicines and largely to universal health coverage. There is, however, limited literature on this subject. Some studies focused on population coverage toward UHC (17), Ghana's progress toward UHC indicators and health service utilization (18), coverage of health services toward UHC (19), and essential medicines in UHC (20).

Examining how and why a country prioritizes and implements pharmaceutical reforms tends to show complex processes and myriad efforts made toward improving access to medicines. This study examines factors that enabled the prioritization of access to medicines reforms and the implementation approaches and how these reforms contributed to universal health coverage in Ghana. This study, specifically, seeks to answer the following questions: (1) What access to medicines pharmaceutical reform items were prioritized since 1999, when the National Drug Policy was developed, and why? (2) What implementation approaches were employed and sustained and why? (3) How did the reforms influence equitable access to medicines and contributed to universal health coverage?

## Methods

A retrospective longitudinal study of pharmaceutical reform items stated in the National Drug/Medicines Policies (10–12) was conducted for the period from 1999 to 2017. The pharmaceutical reform items prioritized over the years were mapped out, and an analysis of the pharmaceutical reforms items and their influence on access to medicines and contributions toward universal health coverage was carried out using three consecutive policies, namely, using the three consecutive policies, namely the National Drugs Policy (1999), National Drug Policy (2004) and National Medicines Policy (2017) (10–12), Health Sector Program of Work (21–25), and National Health Policy (26), Ghana's Roadmap for Attaining Universal Health Coverage (16), National Community-Based Health Planning and Service (CHPS) policy (27), national drug policy implementation evaluation reports, and quantitative data. The gray literature and health sector reports were identified from the Ghana MoH website and Pharmacy directorate office and research articles from Google Scholar. The search terms were “national medicine/drug policy reforms”, “pharmaceutical reform”, “national access to medicine”, “access to medicine agenda”, “national medicines laws”, “UHC and medicines”, “rational selection of medicines”, “rational use of medicines”, “affordable medicines pricing”, “essential medicines list”, standard treatment guidelines”, “national medicines priority”, “sustainable financing for medicines”, “supply chain systems”, and “medicines regulatory systems”. The search year ranged from 1988 when the first essential drug and national formulary with therapeutic guideline was developed in Ghana to 2022. Table 1 summarizes the review guideline. The quantitative data on universal health coverage and access to medicines indicators were sourced from databases maintained by World Health Organization (WHO), the SDG tracker organization, and the Institute for Health Metrics and Evaluation (IHME).

**Table 1:** Literature review guideline.

Objective	To synthesize evidence on access to medicines reforms and its prioritization enabling factors and implementation approaches To synthesize evidence on how the access to medicines reforms contributed to access to medicines and universal health coverage	
Research questions	(1) What access to medicines pharmaceutical reform items were prioritized since 1999, when the National Drug Policy was developed, and why? (2) What implementation approaches were employed and sustained and why? (3) How did the reforms influence equitable access to medicines and contributed to universal health coverage?	
Search strategy	Inclusion	National medicine/drug policy reforms. Health sector policies. National access to medicine. National development policies, health sector policies, and laws aligned to national access to medicines agenda. Gray literature including printed copies of national drug/medicine policies and laws; documents on Ministry of Health and agencies websites; peer-reviewed journals. Language: English
	Exclusion	Exclude countries other than Ghana
	Timeframe	1988–2022
Data source	Institutional website and Office Google Scholar	Ministry of Health (Pharmacy Directorate) National Health Insurance Authority Peer-reviewed literature

### Data analysis

**Figure 1:**
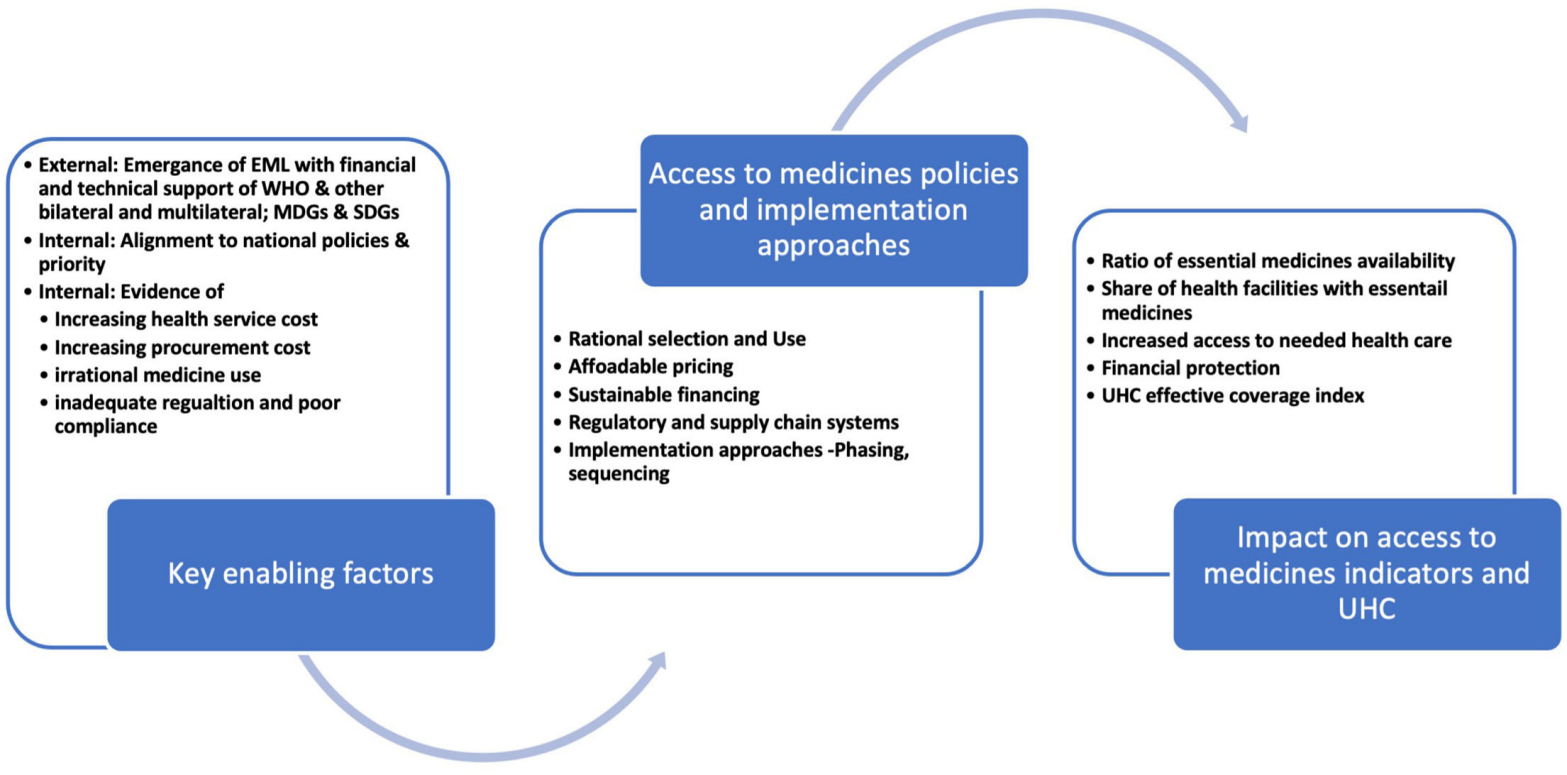
Analytical framework.

Drawing on the WHO's access to medicine dimensions (i.e., rational selection, affordable prices, sustainable financing, and reliable health and supply system) (1) and the universal health coverage dimensions —access to the needed healthcare and financial protection—an analytical framework (Figure 1) was developed to guide the analysis of factors that seem to have played significant roles in the access to medicine policy prioritization, the implementation approaches, and the ways the reforms contributed to universal health coverage. The National Drug/Medicine Policies objectives for 1999, 2004, and 2017, policy items listed, and their alignments to other national policies were mapped for trends. The literature reviewed and the themes based on the analytical framework are summarized in Table 2.

**Table 2:** Summary of literature reviewed and key themes.

**Title/year/source/ reference**	**Theme based on the analytical framework**
Access to Essential Medicines in Ghana: A Survey of Availability of Children's Medicines in Medicine Outlets in the Ashanti Region, 2016 (39)	Availability of essential medicines
Annual programme of work, 2004, MoH Website (56)	Internal factors: Alignment to national policies and priority
Annual programme of work, 2005, MoH Website (57)	
Annual programme of work, 2010, MoH Website (25)	
Assessing approaches to improve adherence to Standard Treatment Guidelines by health professionals in selected regions in Ghana. MoH (Pharmacy Directorate) (32)	Alignment to access to medicine policies—Standard Treatment Guidelines
Assessment of the implementation of the Ghana National Drug Policy 2004–2013, and Proposed policy directions for 2014–2020. MoH (Pharmacy Directorate) (58)	Access to medicine policy assessment
Essential Drugs List and National Formulary with Therapeutic Guidelines, (1988, 1993, 1998) MoH. (59–61)	Access to medicine policy: Rational selection and use
Ghana National Drugs Policy, 1999/Pharmacy Directorate (12)	Access to medicine policies • Rational selection and use • Affordable pricing • Sustainable financing • Regulatory and supply chain systems Internal factors: Evidence of • Increasing health service cost • Increasing procurement cost • Irrational medicine use • Inadequate regulation and poor compliance
Ghana National Drugs Policy, 2004/Pharmacy Directorate (11)	
Ghana National Medicines Policy, 2007, MoH Website. (10)	
Ghana's Roadmap for Attaining UHC 2020-2030/MoH (16)	Internal factors: Alignment to national policies and priority
Health Sector 5-Year Programme of Work, 1996/ MoH (62)	Internal factors: Alignment to national policies and priority
Independent review: Health Sector Programme of Work 2007, 2008, MoH (63)	Internal factors: Alignment to national policies and priority
Institute for Health Metrics and Evaluation. (42)	UHC effective coverage index
Medicines Prices in Ghana, 2008./MoH (34)	Access to medicine policy implementation: Affordable pricing. Medicines availability
Mid-Term Review of the Implementation of the Ghana National Medicines Policy 2017. 2021. MoH (Pharmacy Directorate) (64)	Access to medicine policy assessment
National Community-based Health Planning and Service Policy 2010. MoH, (27)	Internal factors: Alignment to national policies and Priority
National Health Policy 2007/MoH (26)	Internal factors: Alignment to national policies and priority
Pharmaceutical Availability across Levels of Care: Evidence from Facility Surveys in Ghana, Kenya, and Uganda, 2012 (40)	Availability of essential medicines
Procurement Reforms in Ghana 2002 (38)	Access to medicine policy: Regulatory and supply chain systems
Public social policy development and Implementation: a case study of the Ghana National Health Insurance Scheme, 2008, (35)	Financial protection
Standard Treatment Guidelines and Essential Medicines List (2000, 2004, 2010, 2017) MoH (28–31)	Access to medicine policy: Rational selection and use
Use of evidence and negotiation in the review of STG. 2019. (8)	Rational selection of medicines
Value Added Tax (VAT) Exemptions (Amendment) 2017	Access to medicine policy: Medicine pricing Alignment to national policies and priority
VAT Exemptions Regulations 2015	Access to medicine policy: Medicine pricing Alignment to national policies and priority
WHO Essential Medicines List 30^th^ Anniversary/WHO (33)	External factors: Emergence of EML with financial and technical support from WHO

Data on rational selection and use, affordable price, sustainable financing, reliable regulatory and supply system prioritization factors, implementation approaches, and ways these reforms may have contributed to universal health coverage were documented and analyzed. Further analysis involved mapping and categorizing external and internal factors to the identified access to medicine reforms and potential contribution to universal health coverage. The themes in the analytical framework are mapped to the research questions to provide a structure for the presentation of the results. The study reports on access to medicine policies prioritized over time, potential contribution to universal health coverage, and the implementation approaches.

## Results

### Access to medicine policies prioritized and potential contributions to UHC

Table 3 summarizes the access to medicine policy items, National Drug/Medicines Policy objectives, and alignment with other national policies.

**Table 3:** National medicine policy year and objectives, reforms, and alignment with other national policies.

**National drug/medicine policy year and objective**	**Reform dimension and policy items**				**Alignment to other national policies and plans**
	**Rational selection and use**	**Affordable price**	**Sustainable financing**	**Reliable health and supply system**	
**1999:** “*to make essential drugs available and accessible to the population and to ensure the safety, efficacy, and the quality of drugs and their rational use by prescribers, dispensers, and consumers”*	Rational drug use, Drug selection and registration, and Drug advertising and promotion	Local manufacture	Drug financing	Drug storage and distribution	• Essential drug list and formulary with therapeutic guidelines (1988) distributed to health practitioners. • Food and Drugs Law 1992 (PNDCL 305B) and the Pharmacy Act (Act 489) • Ghana National Drugs Programme associated with the development of a National Drug Policy • Vision 2020—growth and development—development of the pharmaceutical sector
**2004:** “*to improve and sustain the health of the population of Ghana by ensuring the rational use and access to safe, effective, good quality, and affordable pharmaceutical products*”	Drug Selection Rational Drug Use	Local manufacture of pharmaceutical and traditional medical products Generic policy	Drug Financing	Quality Assurance	• Health Sector Programme of Work 2nd • Public Procurement Act of 2003 (Act 663) and (Act 914)
**2017:** “*to ensure universal, equitable and sustainable access to priority, efficacious, and safe medicines and other health technologies of acceptable quality for all people living in Ghana and promote their responsible use by healthcare providers and consumers”*	Selection of essential medicines and health technologies Rational Medicine Use Health technology assessment	Medicine Pricing VAT exemptions for local manufacturing	Medicine Financing	Quality assurance of pharmaceuticals	• Sustainable Development Goals (SDGs) toward Universal Health Coverage (UHC), SDG 3. • Ghana Shared Growth and Development Agenda 1 and 2 for wealth creation. • National Health Policy, Creating Wealth through Health, 2007. • Ghana Health Sector Medium Term Plan, 2010–2013 and 2014–2017

#### Rational selection and use of medicines

Rational selection and use of medicine policies have been prioritized over the years to improve patient management practices including diagnosis, prescribing, and dispensing because of evidence of irrational use at all levels within the health sectors (10–12). The main policy items prioritized include the following: First, the national standard treatment guideline (STG) with essential medicines list (EML) (28–31). The standard treatment guidelines and the accompanying EML sought to guide healthcare workers at all levels of care in selecting their treatment options for both adults and children, designing institutional medicines lists, and the procurement of medicines, thus informing the availability and use of essential medicines. To promote the use of the STG and EML, the Ministry of Health over the years organized workshops on the use of the STG and EML and the rational use of medicines for healthcare practitioners (12, 32). For example, the MoH pharmacy directorate held 10 training workshops in all regions between 2016 and 2019 on the use of STG and EML. Health professionals spanning from the public-, private-, and faith-based sectors were trained (32). The EML concept was introduced, promoted, and supported by the World Health Organization to improve access to medicines, and this is a global priority (6, 33). The first national treatment guidelines and medicine list was published in 1988 and revised over the years with the seventh edition published in 2017 (8). Accurate diagnosis through the use of standard treatment guidelines, rational prescription, and the use of medicines is an integral part of quality health service provision and aligns with Ghana's Roadmap for Attaining Universal Health Coverage (16). Over the years, there has been reported improvement in the rational use of medicines; in 2004, the MoH reported a reduction in the average number of drugs prescribed per outpatient encounter from 4.6 to 3.7. In addition, the proportion of outpatients receiving antibiotics and injections has reduced from 54 and 38% to 42 and 33%, respectively (11). Second, the medicine advertisement and promotion policy is prioritized to ensure that the advertising and promotion of medicines are of high professional standards and conform with the requirements of the Food and Drugs Law 1992 (PNDCL 305B) and subsequently the Public Health Act 2012 (Act 851). Advertising and promotion of non-over-the-counter medicines are restricted to only professional publications (12). Third, health technology assessment is prioritized and aligned with Ghana's Roadmap for Attaining Universal Health Coverage. As noted in Ghana's Roadmap for Attaining Universal Health Coverage, health technology assessment is to ensure appropriateness and value for money during the medicines selection process (16). Health technology assessment seeks to support evidence-based decisions around the selection of medicines for use and reimbursement through a process that summarizes the medical, social, economic, and ethical issues related to the use and selection of medicines in a systematic, transparent, and robust manner (10).

#### Affordable price

Access to medicine policies geared at promoting affordable medicines were prioritized over the years because of the increasing cost of medicines with expanding healthcare provision (10–12). Medicines are estimated to constitute 60–80% cost of healthcare, tax tariffs and duties, and markups along the supply chain significantly contribute to the final prices (11). The main policies were prioritized and targeted at cost buildup along the supply chain to provide financial protection, and these align and contribute to universal health coverage (16). First, tax exemptions for pharmaceuticals, i.e., selected active pharmaceutical ingredients, excipients, packaging materials, and selected imported essential medicines. Second, some essential medicines were “ring-fenced” for only local manufacturing to encourage local manufacturing of essential medicines and guarantee market. Third, bulk procurement of medicines through framework agreements to ensure the best-priced and economies of scale. Medicines with high financial impact and high supply risk such as program medicines are to be aggregated, procured, and managed centrally (10). Fourth, generic policy—to ensure affordability in the public sector, medicines are to be procured and prescribed per the EML and in generic (non-proprietary) names only (12). A 2008 medicine use survey found that 87.5% of public sector prescriptions are based on the EML (34). Additionally, a prescription audit across selected regions conducted by the Ministry of Health showed that 98% of medicines prescribed for uncomplicated hypertension were from the first-line recommended medicines in the STG and EML, and 83% of medicines prescribed for severe malaria were in line with the STG and EML (32).

#### Sustainable financing

To promote access to medicines, sustainable financing policies that dealt with increasing funding for essential medicines, expanding health insurance, and the reduction of out-of-pocket expenditures were prioritized. These policies aligned with Ghana's Roadmap for Attaining Universal Health Coverage to ensure a constant flow of medicines and prevent stock-outs across all levels within the health sector. Financing options for essential medicines procurement included the following: First, the government-led medicine financing, where the MoH centrally procures essential medicines; second, government and donor partnership financing; the MoH with support from development partners centrally procures antiretroviral, psychotropics, family planning commodities, and vaccines. Most of the antiretroviral and tuberculosis (90%) are donations and/or products received through the voluntary pooled procurement mechanism from Global Fund and the Global Drug Facility, the United States Agency for International Development—President's Emergency Plan for AIDS Relief, and the Global Alliance for Vaccines and Immunizations (10); third, drug revolving funds in health facilities. Internally generated funds from the sales of medicines are deposited in the designated Bank account and used for medicines procurement to ensure a constant supply of medicines (11); and fourth, the National Health Insurance Scheme (NHIS). NHIS was introduced in 2003 to reduce catastrophic expenditure, especially for the poor and vulnerable (35–37), and as reported in 2010, over 62% of the population accessed care and essential medicines through the NHIS (10).

#### Reliable health and supply system

This dimension focuses on integrating medicines in health sector development and policies, creating an efficient supply chain, and assuring the quality of medicines through regulatory control. Over the years, essential medicines issues are captured in national policies with goals to improve access. For example, the MoH and its stakeholders recognized the importance of procurement in its Medium-Term Health Sector Strategy for Ghana 1997–2001 (38). The supply and management of medicines were rationalized through central and regional medical stores to improve medicine distribution and management at all levels of healthcare delivery (11). This was because of inadequate medicine supply management procedures, and unsuitable and insufficient distribution, and storage facilities, often resulting in increased procurement costs and losses (11). In the public sector, health facilities are necessary to procure the best-priced and quality medicines following procurement laws. Medicines are to be procured centrally, and if the central and regional medical stores are unable to supply them, they can be purchased from the private sector (11). Logistics management information systems are in place to support the quantification and planning, ordering, and tracking of medicines and the availability of medicines at the point of dispensing to the patient (16).

In addition, quality assurance policies were prioritized because of increasing evidence of under-developed machinery to ensure the enforcement of existing laws and regulations resulting in poor compliance (11). The MoH through the Food and Drugs Authority established a National Quality Control Laboratory (NQCL) to test medicines through the supply chain and ensure that only safe and effective medicines are supplied to consumers (11). Also, all medicines for use in Ghana are to be registered by the Food and Drugs Authority. Additionally, a Ghana National Drugs Programme (GNDP) was initiated in 1997 to coordinate the development and implementation of national medicine policies (12). The Ghana National Drugs Programme supported initially by the Dutch government funded activities in both the Food and Drugs Authority and the Pharmacy Council to improve the regulation of medicines and pharmacy practice and promote efficiency in the health sector regulation (11).

Reporting on the access to medicine policies prioritized and their potential contribution to universal health coverage, there were limited quantitative data on the impact of direct improved access to medicines and actual contributions to universal health coverage in Ghana. Some studies focused on the availability of medicines and prescriptions based on the EML and provided information on how accessible medicines were for healthcare services in selected regions and facilities. For example, a 2016 study conducted in 27 districts in the Ashanti region on the availability of 42 children's essential medicine contained in the STG found an overall average availability of 41.3%, while ferrous sulfate syrup (95%), albendazole suspension (90%), and paracetamol syrup (88.8%) had the highest availabilities (39). Another study in 2012 assessed the availability of 50 essential medicines in over 200 facilities in Ghana, Kenya, and Uganda and found that it ranged from 44% in referral hospitals to 16% in community health posts in Ghana. The study further noted that essential medicines availability in Ghana was generally better than that in Uganda and Kenya (40). These studies may not be nationally representative; however, the median availability of selected generic medicine data for Ghana is not available on the WHO Global Health Observatory data repository (41). Nevertheless, we can safely infer that the identified access to medicine policies contributed to the universal health effective coverage index which represents health service coverage across the population. UHC effective coverage index for Ghana has increased from 29.5 in 1990 to 41.6 in 2010 and to 49.1 in 2019 (42).

### Implementation approaches of access to medicine policies

Two main implementation approaches were identified: sequencing and phasing. In this study, the sequencing approach refers to policies implemented and sustained in succession and phasing refers to policies implemented in a distinct period to cover a specific population or type of medicine. For example, the rational selection of medicine policies is implemented in a sequencing approach, because as recommended by the World Health Organization, these policies should be regularly reviewed. The first Essential Drugs List and National Formulary with Therapeutic Guidelines designed for use in 1988 has been revised in 1993, 1996, 2000, 2004, 2010, and 2017 and implemented over years in succession. The MoH through the Ghana National Drugs Programme coordinated the design and implementation of the guidelines and medicines list. The MoH usually with financial and technical support from development partners constantly organizes workshops and meetings to promote the rational use of medicines at all levels. Again, the national health insurance scheme reimbursement medicines list which aligns with the national EML is implemented in succession, as the National Health Insurance Authority together with stakeholders regularly review and implement the list. In addition, the Food and Drugs Authority constantly regulates medicine quality through its National Quality Control Lab.

However, some access to medicine policies was phased out to target specific medicines. For example, the bulk procurement through the framework contracting agreement focused on high-volume, high-risk essential medicines and was implemented in 2018, 2019, and 2021. Again, the tax exemptions for pharmaceutical implementation were phased out. Tax exemptions were implemented for active pharmaceutical ingredients, excipients, and packaging materials for some selected essential medicine locally manufactured in 2015 (43). The tax exemption was later implemented for some selected imported medicines in 2018 (44).

## Discussion

This study explored access to medicine policies prioritized since 1999 when the first national drug policy was designed, the potential contribution of these policies to universal health coverage, and the implementation approach employed. The study highlights the influence of three main factors: (i) evidence, (ii) financial and technical support, and (iii) national and global policies on the prioritization and implementation of the identified access to medicine policies over time. Evidence of irrational use of medicines, increasing medicines cost, supply chain challenges including increasing procurement cost and losses, and inadequate enforcement of medicine regulatory laws influenced the government's decision to prioritize and sustain access to medicine policies over time. Evidence of challenges within the health sector influencing policy prioritization is not new, and this is documented in the literature (45–47). The availability of financial and technical support either from the government or donors also influenced the prioritization of these policies. Donors and government over the years supported the design, review, and implementation of the standard treatment guidelines and essential medicines list, and as a result, these policy items are sustained and prioritized in the national medicine policy (8). The government of Ghana and donor financial support co-existed and influenced the prioritization and implementation of the access to medicine policies, and this is also noted in a scoping review of the health policymaking process in sub-Saharan Africa (48). The finding of donor influence in national policy prioritization is similar to the study on donor influence on national health policymaking in Cambodia and Pakistan (49). The control of financial resources and provision of technical expertise was the most commonly identified route by which donors influenced policymaking processes (49, 50). To fulfill national and global goals, some access to medicine policies was prioritized and sustained. For example, the promotion of local manufacturing of essential medicines aligned with a national goal—Vision 2020, which sought to develop the pharmaceutical sector and affordable medicine pricing and also aligned with the Millennium Development Goal 8E, i.e., “…. *provide access to affordable essential drugs in developing countries”*. The alignment of national policies to global goals and target is usually facilitated by international organizations (51). The access to medicine policy implementation approaches reflects the nature of the policies and also the availability of human and financial resources. Some prioritized policies were sustained and constantly revised, while others were only implemented in distinct periods.

### Access to medicines policies and contribution to UHC

As noted earlier, there were limited Ghana-specific data on access to medicines indicators and the impact of direct improved access to medicines and actual contributions to universal health coverage. There seems to be less priority for data collection and reporting on access to medicine indicators, and this phenomenon is also reported by others (52, 53). The Millennium Development Goals 2015 report also notes the lack of data for indicators of access to medicines and the limited number of surveys undertaken especially between 2007 and 2014 in low-income and lower-middle-income countries (54). Policies promoting medicine affordability and financing are designed and implemented; however, the intended and unintended effects usually in the form of indicators are not fully documented, and sometimes, there are limited data (20). Additional data on medicine expenditure in many countries are limited and, if available, lack adequate details on the types of medicines procured and the degree of access by the population (3). Notwithstanding, the policies targeting rational selection and use, affordable price, sustainable financing, and reliable regulatory and supply system have arguably contributed to the UHC dimension, i.e., access to the needed quality healthcare and financial protection in Ghana. Rational selection and use of medicines policies through improving prescription and dispensing practices, creating an efficient supply chain, and assuring the quality of medicines through a regulatory control system seem to have contributed to improving the general quality of health services. As noted by Ozawa et al. (55), the medicine quality assurance system contributes to reaching universal health coverage goals by ensuring the quality of essential medicines helps deliver effective and safe treatments. In addition, healthcare savings made when quality medicines are used can be reinvested toward universal health coverage (55). Again, the medicine pricing policies and sustainable financing initiatives for medicine may have also contributed to financial protections, especially for the poor and vulnerable.

#### Policy implications

This analysis suggests three policy lessons and implications. First, access to medicine policies targeted at promoting rational medicine selection and use, regulating medicine pricing, improving sustainable medicine financing, and the regulatory and supply chain systems arguably contributed to the attainment of universal health coverage and must be sustained. Second, due to limited data on access to medicines indicators, it is difficult to quantify the policies' impact on universal health coverage and analyze the trend over time. Third, universal health coverage is on both national and global agendas, and therefore, there is a need to constantly galvanize the interest of policy actors toward data collection and reporting indicators for access to medicines.

#### Limitations

First, secondary data were analyzed, and while this may be extensive, primary data sources may have provided additional information. Second, there were limited quantitative data for indicators of access to medicines and their impact on universal health coverage. Despite these limitations, this analysis provides information on access to medicine policies prioritized and how they potentially contribute to financial protection and access to quality healthcare service in Ghana. Future research is recommended in the following areas: evaluation of access to medicine policy implementation across all levels of care and the roles of pharmaceutical players in the implementation of access to medicine policies in Ghana.

## Conclusion

Health sector evidence, financial and technical support, and alignment to national and global policies and goals were the main enablers to prioritization and implementation of access to medicine policies. These policies have been sustained and implemented over time. Although there were limited quantitative data to quantify the effect of the access to medicine policies on universal health coverage, it can be reasonably assumed that, in Ghana, the access to medicine policies has contributed to financial protection and improved access to quality health service.

## Data availability statement

The original contributions presented in the study are included in the article/supplementary material, further inquiries can be directed to the corresponding author.

## Author contributions

AK conceptualized the study, collected and analyzed data, and wrote and reviewed the manuscript.
